# Integrating Protein Engineering and Bioorthogonal Click Conjugation for Extracellular Vesicle Modulation and Intracellular Delivery

**DOI:** 10.1371/journal.pone.0141860

**Published:** 2015-11-03

**Authors:** Ming Wang, Sarah Altinoglu, Yuji S. Takeda, Qiaobing Xu

**Affiliations:** Department of Biomedical Engineering, Tufts University, Medford, Massachusetts, United States of America; George Mason University, UNITED STATES

## Abstract

Exosomes are small, cell-secreted vesicles that transfer proteins and genetic information between cells. This intercellular transmission regulates many physiological and pathological processes. Therefore, exosomes have emerged as novel biomarkers for disease diagnosis and as nanocarriers for drug delivery. Here, we report an easy-to-adapt and highly versatile methodology to modulate exosome composition and conjugate exosomes for intracellular delivery. Our strategy combines the metabolic labeling of newly synthesized proteins or glycan/glycoproteins of exosome-secreting cells with active azides and bioorthogonal click conjugation to modify and functionalize the exosomes. The azide-integrated can be conjugated to a variety of small molecules and proteins and can efficiently deliver conjugates into cells. The metabolic engineering of exosomes diversifies the chemistry of exosomes and expands the functions that can be introduced into exosomes, providing novel, powerful tools to study the roles of exosomes in biology and expand the biomedical potential of exosomes.

## Introduction

Exosomes are small vesicles (40–100 nm in diameter) that are continuously secreted from cells.[1, 2] They carry many different proteins and a significant amount of genetic information that reflects the molecular profiles of their parent cells. In recent years, exosomes have been identified as essential mediators for intercellular communications by transferring a variety of biological signals between cells.[3] Exosome-mediated intercellular transmissions directly alter the functional states of the recipient cells and regulate a diverse range of physiological and pathological processes. Because of these roles, exosomes have emerged as novel biomarkers for disease diagnosis and as biological agents for therapy.[4–6] For example, tumor-derived exosomes contribute to the formation of niches that promote tumor growth and metastasis.[7] Therefore, profiling of the molecular information carried by tumor-secreted exosomes has the potential to be used in cancer diagnosis. Additionally, owing to the high capacity and efficacy of exosomes in exchanging proteins and genes between cells, exosomes have been used as novel nanocarriers for macromolecular drug delivery.[8] For example, loading small-interfering RNA (siRNA) into exosomes for delivery has allowed researchers to silence genes in a highly site-specific manner.[9, 10] The emerging biomedical applications of exosomes require that the molecular compositions of exosomes can be appropriately modulated and engineered to produce exosomes with desired functionalities.[11] Electroporation and genetic engineering[12] have been shown to be the most effective approaches for modifying and integrating new components into exosomes. However, the complicated electroporation and genetic fusion process needed to modulate exosome composition can compromise the efficacy of these exosome engineering approaches. Thus, there remains a great need for powerful yet convenient chemical tools to precisely modulate and diversify exosome component displays to advance and broaden the biomedical potential of exosomes.

**Fig 1:**
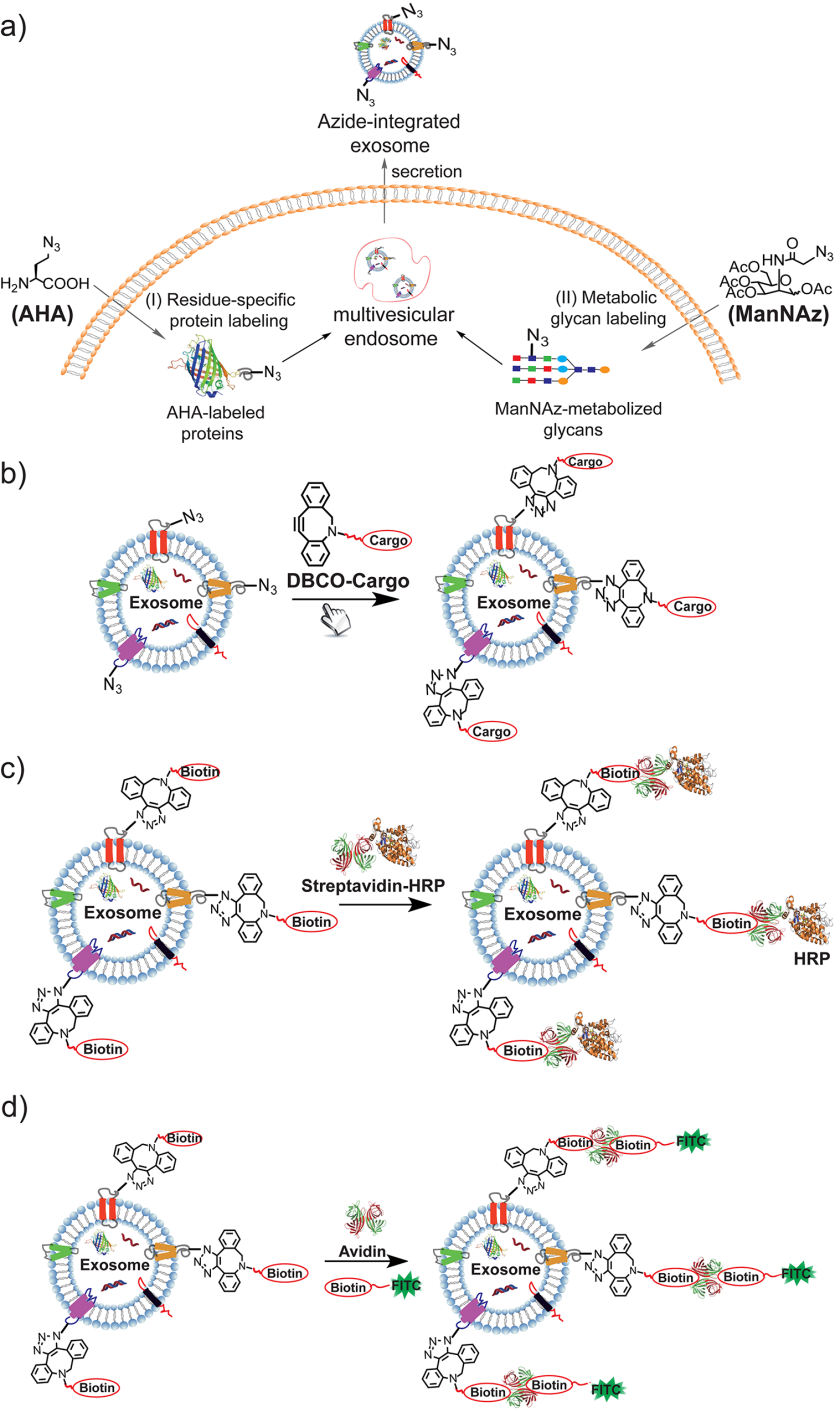
a) Metabolic labeling of newly synthesized proteins and glycans for exosome composition modulation; b) Bioorthogonal click conjugation for exosome functionalization; c) Streptavidin-HRP conjugation to biotinylated exosome; d) Biotin-FITC encapsulation to biotinylated exosome in the presence of avidin.

Here we report an easy-to-adapt and highly versatile methodology to modulate exosome compositions and introduce new functions into exosomes by altering the metabolic processes of exosome-secreting cells. Exosomes are formed inside cells in various multivesicular bodies (MVBs) and are secreted when these MVBs fuse with plasma membranes. A variety of membrane fusion and transport proteins, such as tetraspanins (CD63), lysosomal protein (Lamp2b), and annexin, are integrated into exosomes during the exosome formation and secretion processes.[1] Therefore, we hypothesized that labeling the proteins of exosome-secreting cells using new chemical processes could simultaneously transfer the labeling information onto exosomal proteins, providing an effective way to modulate exosome composition and function. Our strategy combines the metabolic labeling of newly synthesized proteins or glycan/glycoproteins of exosome-secreting cells with chemically active azide groups and bioorthogonal click conjugation to modify and functionalize exosomes (Fig 1). The metabolic engineering and labeling approach conveniently and non-invasively introduces azides onto exosomes, thereby avoiding the need for complicated genetic fusion processes or the harsh metal ion-catalyzed conjugation involved in direct chemical conjugation of exosomes.[13]

In this study, we use a residue-specific protein labeling strategy that incorporates non-canonical amino acids into newly synthesized proteins inside cells to label and engineer exosomal proteins.[14] The metabolic labeling of cellular proteins with non-canonical amino acids has previously enabled the global analysis of protein synthesis and localization inside cells;[15] however, its potential for engineering exosome composition has not yet been fully addressed. Here, L-azidohomoalanine (AHA), an azide-bearing amino acid analogue of methionine (Fig 1, Method A), replaced the natural methionine in newly synthesized proteins inside exosomes.[16] The incorporation of AHA introduces unnatural azides into exosomes, providing additional chemically active sites for exosome bioconjugation through azide-involved bioorthogonal reactions (Fig 1).[17] In addition to the metabolization of AHA described above, the integration of azide-containing saccharides into glycans or glycoproteins of exosome-secreting cells provides another metabolic approach to the modification and functionalization of exosomes (Fig 1). A large number of cells, including cancer cells, decorate their surfaces with a dense layer of sialylated glycans, and specific sialic acid-containing glycoproteins have been identified within tumor cell-secreted exosomes.[18] Therefore, the metabolization of azide-bearing saccharides during the glycan biosynthesis process could simultaneously integrate the saccharides into exosomes. In this study, we co-cultured exosome secreting cells with tetraacetylated N-azidoacetyl-D-mannosamine (ManNAz), an azidosugar that can be metabolized into sialic acid,[19] and obtained ManNAz-integrated exosomes in a manner similar to that of AHA-metabolized exosomes.

AHA or ManNAz metabolization incorporates azides into exosomes and enables exosome functionalization via a broad range of azide-involved bioorthogonal reactions, such as azide-phosphine ligation [20] and azide-alkyne cycloaddition “click” chemistry.[17] As a proof of concept, we report the use of the strain-promoted azide-alkyne click reaction (SPAAC) between dibenzobicyclooctyne (DBCO) and azide to conjugate exosomes with more extensive functions (Fig 1).[21] SPAAC has previously been widely used to study the metabolic biosynthesis of biomolecules in real time in living systems owing to its bioorthogonal activity in complicated environments.[22] Here, we show that SPAAC between DBCO derivatives and azide-integrated exosomes efficiently expanded the functions of exosomes and extended the biomedical uses of engineered exosomes into a novel drug delivery nanoplatform.

## Materials and Methods

### General

L-azidohomoalanine (AHA), tetraacetylated N-azidoacetyl-D-mannosamine (ManNAz), and DBCO-Cy3 were purchased from Click Chemistry Tools (San Diego, CA, USA) and used as received. DBCO-PEG_4_-Biotin used for biotinylation of exosome was obtained from Sigma-Aldrich (St. Louis, MO). The nanoparticle sizes were measured using Brookhaven Zetaplus (Holtsville, NY), TEM of exosomes were imaged using a Tecnai FEG TEM (FEI Tecnai 12 Spirit Biotwin, FEI Company, Hillsboro, OR).

### Preparation and isolation of exosomes

B16F10 cells (purchased from ATCC) were cultured in high-glucose DMEM supplemented with 10% FBS and 1% Penicillin-Streptomycin. To produce AHA-integrated exosomes, B16F10 cells were maintained in conditioned medium composed of methionine- and cystine-depleted DMEM (Invitrogen, Carlsbad, CA) and 10% exosome-free FBS in the presence of 50 μM AHA. For the production of ManNAz-metabolized exosomes, B16F10 cells were fed with 50 μM ManNAz using conditioned medium composed of DMEM and 10% exosome-free FBS. The cells were cultured in different conditioned medium for three days before exosome isolation using differential ultracentrifugation. To isolate exosomes, cell debris was first removed by centrifuging the medium at 2000 × g for 20 min. The microvesicles were then isolated by ultracentrifugation at 10000 × g (12000 rpm) for 45 min. The exosome-containing supernatant was filtered using a 0.20 μm syringe filter (Millipore, Billerica, MA). Finally, exosomes were collected by ultracentrifugation at 100000 × g (40000 rpm) for 150 min. The exosome concentration was determined by BCA protein assay. The pellet of exosomes was resuspended in PBS and stored at −80°C before use.

### DBCO-Cy3 conjugation and flow cytometry analysis of exosomes

100 uL AHA or ManNAz-metabolized exosomes (4 mg/mL of protein) was incubated with 10 μL DMSO solution of DBCO-Cy3 (10 μM final concentration) or 4h at room temperature, followed by ultrafiltration purification using Amicon® Ultra Centrifugal Filters (MWCO = 100 K, Millipore, MA). The exosome concentration was determined by BCA protein assay, the Cy3 concentration was quantified by measuring Cy3 absorption using Nanodrop (Thermo Scientific) and normalizing exosome protein concentration to 1 mg/mL. As a comparison, exosome secreted from B16F10 cells without AHA or ManNAz pre-treatments were mixed with DBCO-Cy3 similarly.

For the flow cytometry analysis of DBCO-Cy3 conjugation of AHA and ManNAz exosomes, 30 μL of Cy3-conjugated exosomes were incubated with 15 μL aldehyde/sulfate latex beads (4 μm in size, Invitrogen, Carlsbad, CA) for 3 h at room temperature. Exosome was conjugated to the beads via the reaction between aldehyde and amine of exosomal proteins, which forms covalent imine bonds. The conjugation of exosomes to the beads was stopped by adding 30 μL FBS, followed by another 60 min. of incubation, the mixed beads were then washed twice using PBS and dispersed in PBS for flow cytometry analysis or exosome surface markers staining. The exosome surface maker, CD63, was stained by incubating exosome-conjugated beads with 3 μL of anti-CD63(Santa Cruz Biotechnology, Inc.) for 2 h, followed by FITC-labeled secondary antibody (Invitrogen, Carlsbad, CA) staining. The beads were washed with PBS for two times before flow cytometry analysis on BD FACScalibur.

### Intracellular delivery of DBCO-Cy3 conjugated exosomes

B16F10 cells were seeded in 96-well plates at a density of 15,000 cells per well 24 h prior to the experiment. At the day of delivery, the cells were treated with AHA-Cy3 or ManNAz-Cy3 exosomes (with 100 nM of Cy3) for 6 h before harvesting for flow cytometry analysis. Free DBCO-Cy3 or a mixture with exosome secreted by untreated B16F10 cells were similarly exposed to B16F10 cells as negative controls. For the CLSM imaging of DBCO-Cy conjugated exosome delivery, B16F10 cells were seeded in CELLview™ Cell Culture Dishes (Greiner Bio One, Inc) at a density of 50, 000 cells per well 24h prior to the experiment. The cells were treated with free DBCO-Cy3, AHA-Cy3, or a mixture of DBCO-Cy3 and control B16F10 exosomes for 6 h, in which 100 nM of Cy3 was exposed to cells. At the end of delivery, the cells were washed three times with PBS before CLSM imaging.

### DBCO-PEG_4_-biotin conjugation of AHA-exosomes

For the biotinylation of exosomes, 100 uL AHA-metabolized exosomes (4 mg/mL of exosomal protein) was incubated with 10 μL DMSO solution of DBCO-PEG_4_-Biotin (1 mM final concentration) for 6h at room temperature, followed by ultrafiltration purification using Amicon® Ultra Centrifugal Filters (MWCO = 100 K, Millipore, MA). The biotin-conjugated exosomes were dispersed in PBS and spotted onto PVDF membranes, followed by overnight incubation in 5% non-fat milk solution, immunoblotting with streptavidin-HRP conjugates (Molecular Probes, Carlsbad, CA), the spots were visualized using ECL system on Syngene G-Box (Cambridge, UK). For negative controls, AHA-exosome without DBCO-PEG_4_-biotin conjugation, or control B16F10 exosomes with and without DBCO-PEG_4_-biotin conjugation were similarly blotted onto PVDF membranes.

### Intracellular delivery of streptavidin-HRP using biotin-conjugated exosomes

Biotin-modified exosome (0.5 mg/mL of protein) was incubated with varied concentration of streptavidin-HRP (with protein concentration increased from 0 to 1.5 μg/mL) for 15 min. before adding to B16F10 cells seeded in 96-well plate. After 6 h of incubation, the culture medium was removed and the cells were washed three times with 1mg/mL of heparin solution. The intracellular HRP activity was assayed by adding 100 μL 3,3′,5,5′-Tetramethylbenzidine liquid substrate (Sigma-Aldrich, St. Louis, MO) per well, followed by additional 30 min. of incubation at 37°C. The absorption of the solutions was then monitored at 655 nm and normalized to blank controls.

### Intracellular delivery of Fluorescein-biotin conjugates using biotin-clicked exosomes

B16F10 cells were seeded in CELLview™ Cell Culture Dishes (Greiner Bio One, Inc) at a density of 50, 000 cells per well 24h prior to the delivery. At the day of experiment, 0.12 μg fluorescein-biotin (Sigma-Aldrich, St. Louis, MO) and 7.5 μg avidin was pre-mixed in 75 μL PBS for 15 min. before adding 25 μL biotion-conjugated exosomes. The mixtures were incubated for another 20 min. before exposing cells. The culture medium was removed 6 h post delivery, cells were washed three times with 1mg/mL of heparin solution before CLSM imaging.

## Results and Discussion

**Fig 2:**
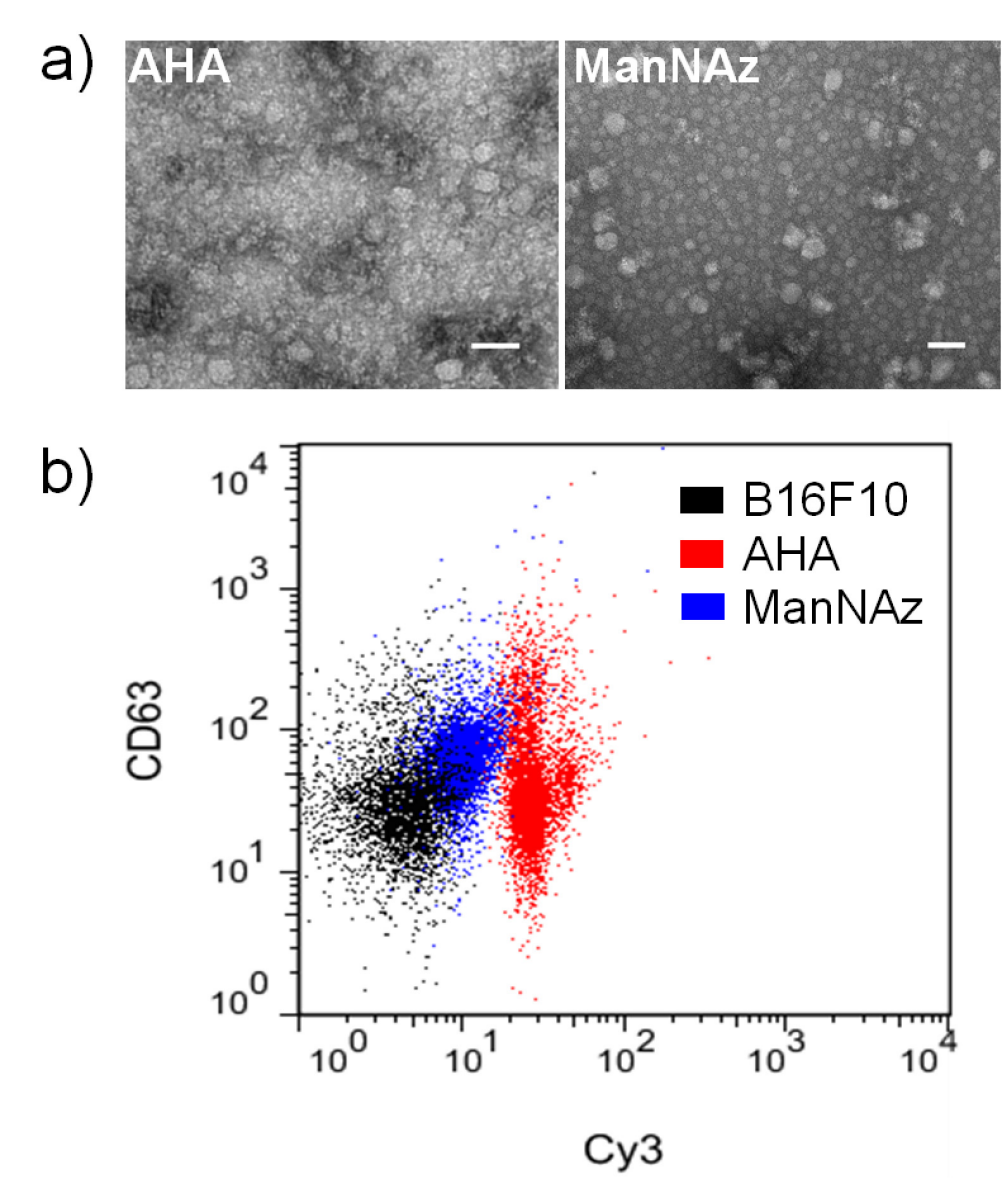
a) TEM images of AHA- and ManNAz-metabolized exosomes. Scale bar: 50 nm. b) Flow cytometry analysis of AHA exosomes, ManNAz exosomes, and control exosomes secreted by untreated B16F10 cells. The exosomes were mixed with DBCO-Cy3 and conjugated to polystyrene beads before staining with anti-CD63.

The metabolized exosomes can be obtained by co-culturing AHA or ManNAz (50 μM each substrate) with exosome-secreting B16F10 cells for three days and then isolating exosomes using differential ultracentrifugation. The diameters of the AHA- and ManNAz-metabolized exosomes are approximately 70 nm and 50 nm, respectively, as measured using dynamic light scattering (DLS) analysis (S1 Table) and transmission electron microscopy (TEM, Fig 2). To confirm the successful incorporation of AHA and ManNAz into exosomes during the cell co-culture process and the efficient click conjugation of these engineered exosomes, we first incubated AHA- and ManNAz-metabolized exosomes with fluorescent DBCO-Cy3 and then quantified Cy3 conjugation efficiency after removing the excess DBCO-Cy3 from the reaction mixtures. The concentration of Cy3 in the AHA-Cy3- and ManNAz-Cy3-clicked exosomes was measured to be 790 nM and 440 nM, respectively, after normalizing the exosome protein concentration to 1 mg/mL. Meanwhile, we did not observe significant exosome size changes after DBCO-Cy3 clicking (S1 Table), suggesting that the azide-integrated exosomes are highly compatible with SPAAC conjugation in terms of retaining exosome integrity. We further conjugated AHA-Cy3 and ManNAz-Cy3 exosomes to polystyrene beads for flow cytometry analysis, using exosomes secreted by untreated B16F10 cells mixed with DBCO-Cy3 as a negative control. Additionally, a representative exosome surface marker protein, CD63,[2] was immunostained to evaluate the effects of our metabolic engineering approaches and click conjugation on preserving exosome integrity. As shown in Fig 2, the beads conjugated with AHA-Cy3 and ManNAz-Cy3 exosomes both had enhanced Cy3 fluorescence intensities compared to the control B16F10 exosomes, whereas no significant differences in CD63 level were observed among the AHA-Cy3, ManNAz-Cy3, and control B16F10 exosomes. The preliminary click conjugation of AHA- and ManNAz-metabolized exosomes with DBCO-Cy3 confirmed the efficient integration of azides into these exosomes, which provides a convenient approach for the functionalization of exosomes.

**Fig 3:**
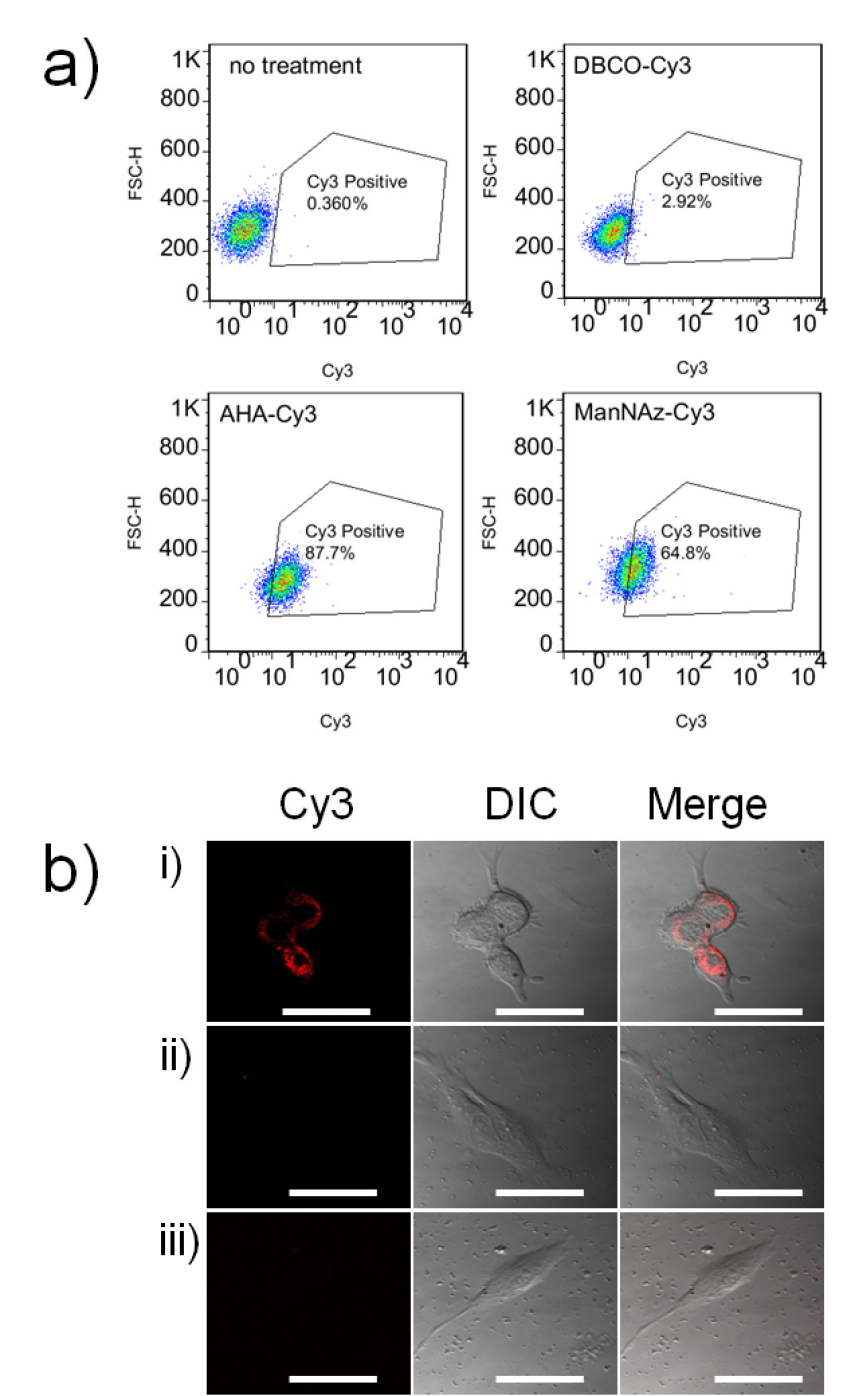
a) Flow cytometry analysis of B16F10 cells following different DBCO-Cy3 treatments; b) CLSM images of B16F10 cells treated with i) AHA-Cy3 exosomes, ii) free DBCO-Cy3, and iii) DBCO-Cy3 mixed with control B16F10 exosomes. Scale bar: 20 μm. DBCO-Cy3 (100 nM) was incubated without and with exosomes (0.5 mg/mL protein) for 3 h before being exposed to cells.

Inspired by the high efficacy of exosomes for transporting biological signals between cells and the efficient integration of azides into AHA- and ManNAz-metabolized exosomes for SPAAC conjugation, we next studied whether these engineered exosomes could click cargos and transport them into cells for drug delivery. To this end, we treated B16F10 cells with AHA-Cy3 and ManNAz-Cy3 exosomes and quantified the fluorescent cells to evaluate the ability of azide-integrated exosomes to transport conjugated Cy3 into cells. Flow cytometry analysis of the B16F10 cells that received different treatments (Fig 3A) indicated that DBCO-Cy3 (100 nM) alone cannot enter cells. However, when the cells were treated with the same concentration of DBCO-Cy3 clicked with AHA- or ManNAz-metabolized exosomes (0.5 mg/mL of proteins), the percentage of fluorescent cells was increased to 87% for AHA-Cy3 and 65% for ManNAz-Cy3 exosome treatments, suggesting that there was efficient uptake of AHA-Cy3 and ManNAz-Cy3 exosomes by B16F10 cells. The intracellular localization of Cy3-conjugated exosomes was further visualized by confocal laser scanning microscopy (CLSM). As shown in Fig 3B, the treatment of B16F10 cells with free DBCO-Cy3 (100 nM) or a mixture of DBCO-Cy3 (100 nM) and control B16F10 exosomes (0.5 mg/mL of proteins) could not deliver Cy3 into cells, whereas the AHA-Cy3 exosome treatment (100 nM Cy3) resulted in significant Cy3 accumulation in the cytosol. Taken together, both the flow cytometry analysis and CLSM study suggest that azide-integrated AHA and ManNAz exosomes could click cell-impermeable molecules and efficiently transport them into cells.

Having demonstrated the ability of azide-integrated exosomes to conjugate DBCO derivatives for intracellular delivery, we extended the SPAAC chemistry of exosomes to a broad range of DBCO derivatives and expanded the functionalities that can be introduced to exosomes. Here, biotin, a small biomolecule that displays a strong binding affinity to avidin or streptavidin, was clicked to AHA exosomes to decorate the exosome with biotin ligands. The biotin conjugation (biotinylation) of exosomes diversifies the exosome functionalities and enables the binding and encapsulation of avidin-fused cargos into exosomes through biotin-avidin interactions. Additionally, in a large number of diseases, cells overexpress biotin receptors; thus, the biotinylation of exosomes has potential for use in targeted imaging and drug delivery. The biotinylation of azide-integrated exosomes was achieved by clicking AHA exosomes with DBCO-PEG_4_-biotin (1 mM), followed by centrifugal purification to remove excess DBCO. The successful biotinylation of AHA exosomes was confirmed using a dot blot assay. AHA exosomes before and after DBCO-PEG_4_-biotin clicking were dot-blotted onto a PVDF membrane, followed by probing and imaging with streptavidin-horseradish peroxidase (HRP) conjugates. As shown in S1A Fig, the biotinylation of only AHA exosomes, but not control B16F10 exosomes, was observed when both exosomes were similarly incubated with DBCO-PEG_4_-biotin. Meanwhile, we did not observe significant variations in the size of AHA exosomes before and after the biotin clicking, as shown by the DLS nanoparticle size analysis and TEM imaging (S1 Table and Fig 1B), suggesting that the biotinylation of the exosomes via SPAAC conjugation did not impair exosome integrity.

**Fig 4:**
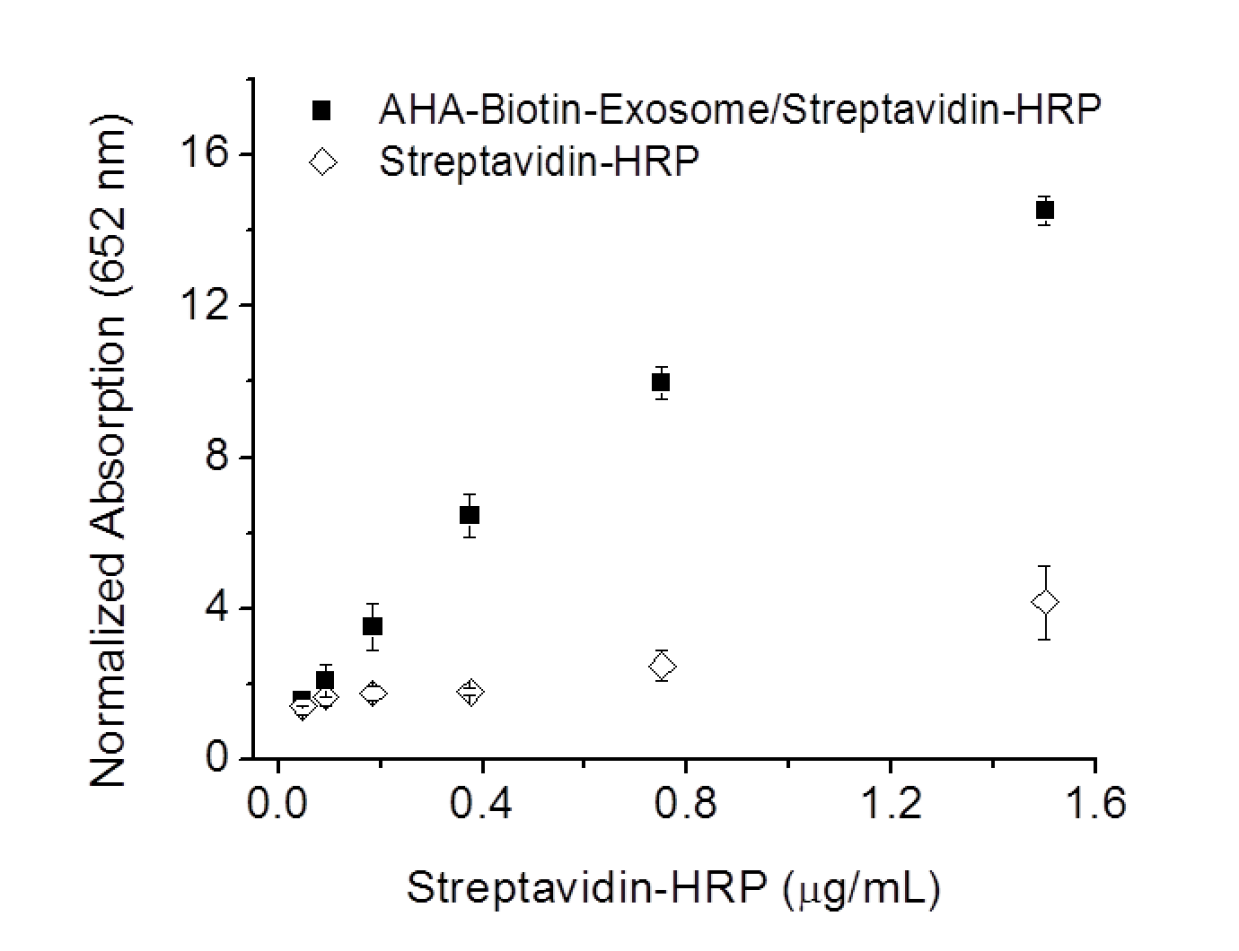
Intracellular delivery of streptavidin-HRP using biotin-conjugated AHA exosomes. B16F10 cells were treated with various concentrations of protein pre-incubated with and without exosomes.

**Fig 5:**
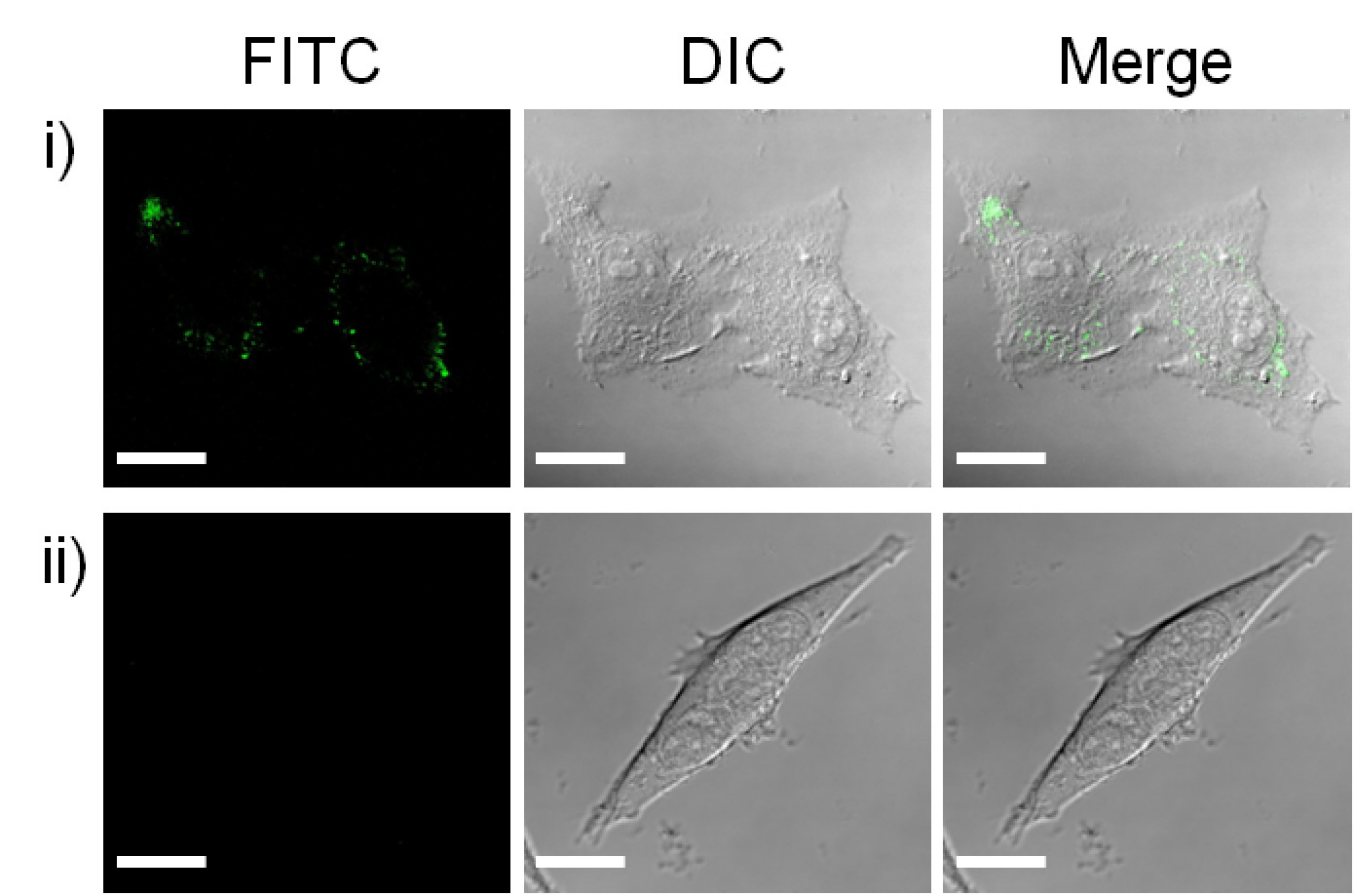
CLSM images of B16F10 cells treated with the biotin-4-fluorescein/avidin complex in the presence (i) and absence of biotin-clicked exosomes (ii). Scale bar: 20 μm.

The biomedical potential of biotin-clicked exosomes was first demonstrated by encapsulating streptavidin-fused proteins into exosomes and transporting these proteins into cells. The intracellular delivery of proteins to replace dysfunctional proteins and modulate cell signaling pathways has been recognized as the most direct and efficient approach for manipulating cell function and treating disease.[23, 24] Here, we demonstrated that biotin-clicked exosomes efficiently encapsulated and delivered streptavidin-HRP into B16F10 cells in an active form. As shown in Fig 4, B16F10 cells treated with streptavidin-HRP alone had very weak HRP activity because naked streptavidin-HRP has low efficiency for entering into cells. However, after treating cells with streptavidin-HRP (1.5 μg/mL) pre-incubated with biotin-modified exosomes (0.2 mg/mL of protein), the intracellular HRP activity was increased up to 6 times compared with treatment with the free protein. Meanwhile, the intracellular HRP activity was enhanced with the concentration of protein increased from 0.1 to 1.5 μg/mL, indicating the efficient delivery of streptavidin-HRP after incubation with biotin-clicked exosomes. Lastly, we demonstrate that the biotin-clicked exosomes were able to deliver biotin-conjugated cargos into cells by complexing with avidin. (Strept)avidin has multiple biotin binding sites; therefore, the biotin-clicked exosomes could encapsulate additional biotin conjugates and form nanocomplexes for intracellular delivery in the presence of avidin. We selected biotin-4-fluorescein as a model cargo to illustrate the concept and ability of biotin-clicked exosomes to deliver biotin conjugates. The pre-incubation of biotin-4-fluorescein (13 μg/mL) with avidin (80 mg/mL), followed by the addition of biotin-clicked exosomes (0.5 mg/mL of protein), formed a nanocomplex that efficiently delivered biotin-4-fluorescein into cells. As shown in Fig 5, significant intracellular accumulation of biotin-4-fluorescein was observed when B16F10 cells were treated with the above nanocomplex, while negligible amounts of biotin-4-fluorescein were delivered when the cells were treated with the biotin-4-fluorescein/avidin complex in the absence of the exosomes. The effective delivery of protein and biotin conjugates using biotin-clicked exosomes highlights the advantages and versatility of azide-bearing exosomes for intracellular delivery, and this strategy should be easily adaptable for the delivery of various avidin-fused or biotin-conjugated drugs.

## Conclusions

In conclusion, we have reported the first example of the metabolic engineering of exosome-secreting cells to introduce new chemistries (active azides) into exosomes in a highly compatible manner. Combined with bioorthogonal click conjugation, our method allows the convenient yet effective modulation of exosome composition and function. These engineered exosomes have further been extended to a novel drug delivery platform by clicking variable cargos into exosomes, including small molecules and proteins. The metabolic engineering strategy that we report here is not limited to azide-bearing amino acids and saccharides; all other substrates that are capable of integrating into multivesicular bodies could be similarly used to functionalize exosomes. The efficient modulation of exosome composition will spur the development of multifunctional exosomes for disease diagnosis and therapy. For example, the incorporation of active target ligands and therapeutics into exosomes will enable the use of novel nanocarriers for targeted drug delivery. The click conjugation of multiple imaging probes into exosomes will lead to an exosome-based multimodal imaging system for disease diagnosis. Our results provide insights into the potential roles of exosomes in biology and clinical therapies.

## Supporting Information

S1 Fig a) Dot-blotting assay of AHA-intergrating exosome with and without DBCO-PEG4-Biotin click conjugation. Exosomes secreted by B16F10 cells without AHA treatment was used as a negative control; b) TEM images of biotin-conjugated exosome. Scale bar: 50 nm.
(TIF)

S1 Table DLS particle sizes of AHA- and ManNAz-integrated exosome before and after click conjugations (TIF)
