# Supplementary figures and images for: Integrating Protein Engineering and Bioorthogonal Click Conjugation for Extracellular Vesicle Modulation and Intracellular Delivery
Source: PLoS One. 2015 Nov 3;10(11):e0141860. doi: 10.1371/journal.pone.0141860 (PMC4631329; doi:10.1371/journal.pone.0141860)

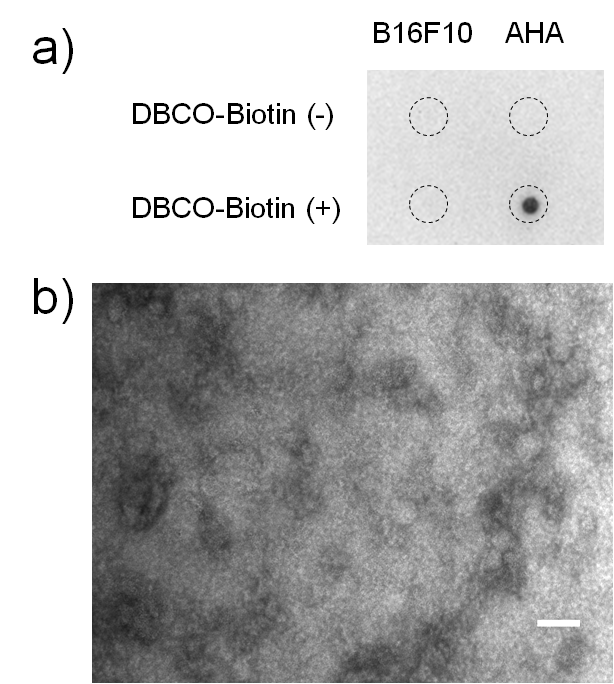

Supplement: S1 Fig — Scale bar: 50 nm. (TIF) [file pone.0141860.s001.tif]

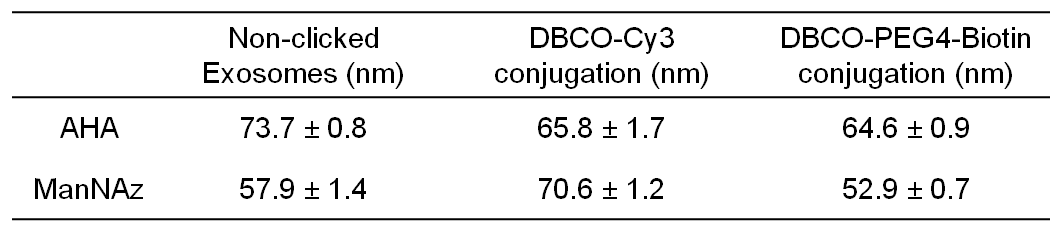

Supplement: S1 Table — (TIF) [file pone.0141860.s002.tif]
